# Early Tube Feeding Improves Nutritional Outcomes in Children with Neurological Disabilities: A Retrospective Cohort Study

**DOI:** 10.3390/nu15132875

**Published:** 2023-06-25

**Authors:** Valeria Dipasquale, Ugo Cucinotta, Angela Alibrandi, Francesca Laganà, Vincenzo Ramistella, Claudio Romano

**Affiliations:** 1 Pediatric Gastroenterology and Cystic Fibrosis Unit, Department of Human Pathology in Adulthood and Childhood “G. Barresi”, “G. Martino” University Hospital, 98124 Messina, Italy; dipasquale.va@gmail.com (V.D.); ugocucinotta@gmail.com (U.C.); fralaga91@gmail.com (F.L.); vinsramis@hotmail.it (V.R.); 2 Statistical and Mathematical Sciences Unit, Department of Economics, University of Messina, 98122 Messina, Italy; angela.alibrandi@unime.it

**Keywords:** BMI, enteral formula, enteral nutrition, gastrointestinal symptoms, tube feeding, weight-for-age

## Abstract

Tube feeding is a life-saving treatment for children with neurological disabilities (ND), who often suffer from malnutrition and feeding disorders. Nonetheless, it is still not widely used. Our aim was to evaluate the outcomes of exclusive tube feeding in a cohort of ND children. All consecutive ND children who started tube feeding at our center within the last 5 years were included in this retrospective study. Weight-for-age, body mass index (BMI), mid-upper arm circumference (MUAC) Z-scores, and symptoms were collected at baseline (V0), 6 (V1), and 12 months (V2) after gastrostomy placement. Fifty children (62% males) were included. The ND-underlying disease was genetic (*n* = 29, 58%), hypoxic-ischemic encephalopathy (*n* = 17, 34%), or metabolic (*n* = 4, 8%). Indications for tube feeding were malnutrition (*n* = 35, 70%), recurrent respiratory infections (*n* = 11, 22%), or both (*n* = 4, 8%). Enteral formulae were polymeric (*n* = 29, 58%), semi-elemental (*n* = 17, 34%), hypercaloric (*n* = 3, 6%), or elemental (*n* = 1, 2%). Homemade blended feed was offered to three children (6%) in addition to the formula. Weight and BMI increased over the study period. Except for constipation, all symptoms (cough, vomiting, and diarrhea) improved at 6 and 12 months (*p* < 0.05). Non-serious complications (*n* = 8; track disruption, granuloma, and skin infection) were observed. Longer disease duration (*p* < 0.001) at the start of tube feeding was associated with the absence of normalization of nutritional status (BMI Z-score > 2 SD) at 12 months. Tube feeding with commercially available enteral formulae should be started as early as possible for better outcomes.

## 1. Introduction

According to the disabilities prevalence estimates among children younger than 18 years reported by UNICEF 2022, a total of 28.9 million or 4.3% (95% CI: 4.1–4.6) of children aged 0–4 years, 207.4 million or 12.5% (95% CI: 11.7–13.3) of children aged 5–17 years, and 236.4 million or 10.1% (95% CI: 9.6–10.6) of all children aged 0–17 years were estimated to have moderate-to-severe disabilities globally [1]. In particular, children with moderate-to-severe neurological disability (ND) often suffer swallowing and/or gastrointestinal complaints and require exclusive tube feeding [2]. Tube feeding indications include optimizing nutritional status and growth and preventing malnutrition; maintaining fluid intake; improving drug compliance; reducing aspiration and complications associated with gastroesophageal reflux disease (GERD); and improving the overall health-related quality of life (HRQoL) of children and their caregivers and families [3]. 

Nutritional rehabilitation is the major indication for tube feeding, and its therapeutic efficacy is measured in terms of nutritional status normalization. Moreover, malnutrition may have a significant impact on respiratory functions. It impairs the activity of the respiratory muscles, the structure of the lungs, the body’s immune defense, and the regulation of the ventilation, predisposing patients to respiratory failure and prolonged artificial ventilation. According to some studies, malnutrition may be a risk factor for some respiratory conditions as asthma and tuberculosis [4,5]. Additionally, malnutrition has been linked to immune system dysfunction (i.e., reduced exocrine secretion of protective chemicals, low levels of plasma complement, and altered gut-barrier function), which may increase mortality [6]. What is most significant is that with proper nutritional rehabilitation, many of the negative effects of malnutrition can be partially reversed. 

The nutritional management of young patients with ND was addressed in the 2017 European Society of Gastroenterology, Hepatology, and Nutrition (ESPGHAN) consensus manuscript, which gave recommendations on the time and modalities of tube feeding [7]. Tube feeding has been shown to be effective in avoiding and/or reversing malnutrition in pediatric ND patients [8]. Tube feeding is a life-saving treatment for children with ND, but it is not yet widely used or is used late with respect to the course of the underlying disease. The aim of this study was to assess the nutritional and clinical outcomes of exclusive tube feeding with enteral formula in a relatively large cohort of ND children.

## 2. Materials and Methods

In this retrospective study, all consecutive ND children who started enteral nutrition through gastrostomy or gastrojejunostomy between 2016 and 2021 at the Pediatric Gastroenterology Unit of the University Hospital of Messina, Italy, were included. Inclusion criteria were age ≤ 18 years; progressive or nonprogressive ND, such as any condition related to the central nervous system, made up of the brain and spinal cord, with impairment of the individual’s language, cognitive, and motor skills [7]; and indication for exclusive enteral nutrition. According to the routine standard of care at our center, children for whom nutritional rehabilitation was clinically indicated underwent PEG placement by standard pull technique without acidic or non-acidic reflux pH measurements. 

At baseline (V0; namely, before gastrostomy placement), 6 (V1), and 12 months (V2) after gastrostomy placement, nutritional parameters (weight-for-age, body mass index, BMI, and mid-upper arm circumference, MUAC, Z-scores), and symptoms (coughing, vomiting, sialorrhea, and constipation) were retrospectively collected from medical records. The standard charts used were the World Health Organization growth charts as per international guidelines. MUAC was registered in children aged ≥2 months, while BMI was registered in those aged 2 to 19 years. Energy needs were estimated starting from the dietary reference intake (DRI) for basal energy expenditure for typically developing children [9,10] then individualized according to mobility, muscle tone, and activity level based on the Gross Motor Function Classification Scale (GMFCS) [11]. For symptoms, parent-proxy reports were relied on when the children were not capable of self-reporting [7]. Oropharyngeal dysphagia was classified according to the Dysphagia Outcome and Severity Scale (DOSS) [12]. 

At baseline, variables including age, gender, and underlying ND-associated disease were also collected. 

Predictive factors for nutritional status normalization (BMI Z-score > −2 standard deviation, SD) and resolution of symptoms at 12 months were investigated. 

The ethics committee of the University Hospital of Messina approved the data collection (study protocol n. 66/21, ethical approval dated 24 November 2021). The study protocol conforms to the ethical guidelines of the 1975 Declaration of Helsinki (6th revision, 2008). Informed consent was obtained from the parents or legal representatives of the children included in the study. 

### Statistical Analysis

The numerical variables were expressed as median values and interquartile ranges (Q1–Q3), and the categorical variables as absolute frequencies and percentages. The non-parametric approach was used since most numerical variables were not normally distributed, as verified by the Kolmogorov–Smirnov test. The Wilcoxon test for dependent samples was applied in order to individuate possible significant differences between three consecutive timepoints (V0 vs. V1, V1 vs. V2, and V0 vs. V2) with reference to numerical variables. The McNemar test was applied for the same purpose, with reference to dichotomous variables. The Spearman correlation coefficient was applied in order to evaluate the correlation between the normalization of the nutritional status (BMI > −2 SD) at 12 months and some variables of interest such as the age at starting tube feeding, the disease duration before starting tube feeding, the level of gross motor impairment according to the GMFCS, the presence of epilepsy, and the ND-underlying disease. The Spearman correlation coefficient was also applied in order to evaluate the correlation between the resolution of symptoms at 6 and 12 months and some variables of interest such as GMFCS, epilepsy, the ND-underlying disease, the type of enteral formula, and the enteral regimen. The point-biserial correlation test was used for analyzing the correlation between numeric variables (age at starting tube feeding and disease duration before starting tube feeding) and a dichotomous one (normalization of the nutritional status at 12 months (yes or no).

Statistical analyses were performed using IBM SPSS for Windows, Version 22 (Armonk, NY, USA, IBM Corp.). *p*-values lower than 0.05 were considered statistically significant.

## 3. Results

A total of 50 children were included (31 males, 62%; mean age at the start of tube feeding: 4.65 years; range: 3 months–16 years). ND-underlying diseases were represented by genetic syndrome (*n* = 29, 58%), hypoxic-ischemic encephalopathy (*n* = 17, 34%), and metabolic syndrome (*n* = 4, 8%). Most children had severely impaired gross motor function (IV-V GMFCS; *n* = 42, 84%) and epilepsy (*n* = 36, 72%). Indications for nutritional rehabilitation (i.e., tube feeding) were represented by malnutrition (*n* = 35, 70%), recurrent respiratory infections (including aspiration pneumonia) (*n* = 11, 22%), or both (*n* = 4, 8%). The characteristics of enteral nutrition are summarized in Table 1.

Weight and BMI significantly increased over the study period (Table 2).

Nearly all of the children (*n* = 48, 96%) experienced one or more symptoms at baseline: coughing, *n* = 37 (74%); vomiting, *n* = 29 (58%); sialorrhea, *n* = 21 (42%); constipation, *n* = 1 (2%). All symptoms included in the analysis significantly improved between baseline and both 6 and 12 months (coughing, *p* < 0.001 and *p* = 0.001, respectively; vomiting, *p* < 0.001 and *p* = 0.001, respectively; sialorrhea, *p* = 0.004 and *p* = 0.002, respectively) according to the McNemar test.

A few mild complications (*n* = 8; track disruption, granuloma, peristomal site infection) were observed over the study period.

**Figure 1:**
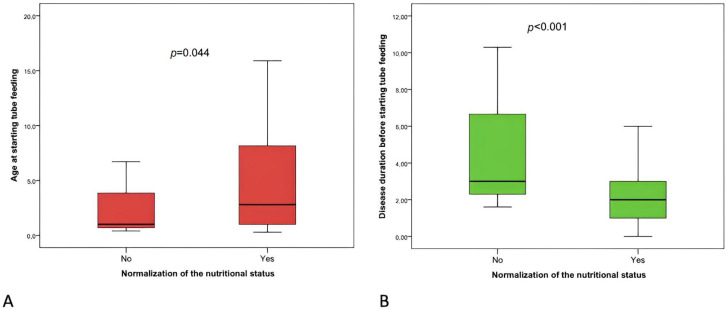
Boxplot of the distribution of the age at starting tube feeding (**A**) and the disease duration before starting tube feeding (**B**) in two groups defined by the normalization of the nutritional status at 12 months (yes or no).

The normalization of the nutritional status at 12 months was achieved in 37 (74%) patients, while it was not achieved in the remaining. The subjects with normalization of nutritional status at 12 months were significantly older at initiation of tube feeding than those in whom nutritional status does not normalize, and those in whom nutritional status did not normalize at 12 months had a significantly longer disease duration (before the start tube feeding) than those in whom nutritional status normalized (Figure 1).

No association was found between the normalization of the nutritional status at 12 months and GMFCS (I-II-III-IV-V), epilepsy (yes/no), or the ND-underlying disease (genetic syndrome/hypoxic-ischemic encephalopathy/metabolic syndrome). Furthermore, no association was found between the resolution of symptoms at 6 and 12 months and GMFCS (I-II-III-IV-V), epilepsy (yes/no), the ND-underlying disease (genetic syndrome/hypoxic-ischemic encephalopathy/metabolic syndrome), the type of enteral formula (polymeric isocaloric/semielemental isocaloric/hypercaloric/elemental), and the enteral regimen (intermittent/boluses/continuous).

## 4. Discussion

A significant proportion of ND children, particularly those with severe and longer-term gross motor impairment and oropharyngeal dysfunction, are considered to be at high risk of poor nutritional status. Tube feeding aims at improving the growth and nutritional status of children with ND-associated dysphagia and malnutrition. Long-term follow-up studies showed that exclusive enteral nutrition via tube feeding is a safe, efficient, and cost-effective feeding technique [8,13,14,15]. One of the first longitudinal, prospective, multicenter cohort studies by Sullivan and colleagues [8] enrolled 57 children with cerebral palsy receiving a gastrostomy with a follow-up of 12 months and showed a significant increase in weight gain and triceps skinfold thickness over the study period. In a more recent study, 38 ND children started exclusive tube feeding with a standard polymeric formula over five years. At baseline, nutritional parameters (body weight, BMI, and triceps skinfold thickness) were ≤5th centile (≤−2.5 *z*-score) in all 38 patients, in comparison with the standards for typically developing children. Nearly all of the children included showed a significant increase in body weight, BMI, and triceps skinfold thickness at 12 months after gastrostomy [16]. In the present study, weight and BMI significantly increased at 6 and 12 months after the start of tube feeding, while no significant improvement was observed in MUAC. This could be explained by the relatively short follow-up since MUAC reflects the amount of muscle mass and this may need longer timelapse to fully recover. The nutritional assessment of ND children is primarily based on low weight and/or low triceps skinfold thickness according to the ESPGHAN guidelines [7], which are time-consuming and require trained staff in this group of children. In children older than 2 years with cerebral palsy, obtaining weight by direct measurement becomes more difficult due to lack of balance and the motor compromise preventing them to stay still on a regular scale, particularly in low- and middle-income countries with limited access to wheelchair adapted scales or even regular scales [17,18]. MUAC, a segmental measure indirectly assessing growth and changes in caloric and protein intake, uses the fat, bone, and muscle areas of the arm as an indirect measure to evaluate body weight [18,19]. Using circumferences, primarily MUAC, in combination with other methods has consistently been recommended for children with ND.

In this cohort, different types of enteral formulae were used. Not surprisingly, the type of enteral formula in children with ND did not predict the normalization of nutritional status. The choice of enteral formula comes from different factors, including the patient’s age, nutritional requirements, type of enteral access, and child’s tolerance to feeds [7,20,21]. A standard (1.0 kcal/mL) polymeric age-appropriate formula including fiber is recommended as the initial enteral feed after 1 year of age [7]. A high-energy density formula (1.5 kcal/mL) including fiber, dietary supplementation with glucose polymers, and/or long-chain triglycerides is suggested for children with increased caloric needs or poor tolerance of large volumes of feed. Elemental formulae may be attempted in cases of GERD, gagging, and retching [7]. However, in this study, the type of formula did not predict symptom resolution by the end of the study. This may show that when that child tolerates the formula and is stable, his or her condition can improve. Although standard commercial formulae are nutritionally complete and are currently the most commonly used, real food-based enteral feeding, including blenderized tube feeds and formulae containing real food, is becoming increasingly popular among parents and caregivers of patients on long-term enteral nutrition [19]. They can help improve gastrointestinal symptoms such as constipation, reflux, and choking [22]. However, in our center, ND children on real food-based formulae had less than a 12-month follow-up, so they were excluded from the present cohort study.

It Is widely known that tube feeding via gastrostomy is safe for the patient. In the prospective study by Sullivan and colleagues [8], an infection rate of 59%, leakage of 30%, and granuloma of 42% were reported. Only one serious complication (gastric leakage and peritonitis) occurred in one of the children who had undergone simultaneous laparoscopic gastrostomy and fundoplication. In the relatively recent retrospective cohort study, which included 38 pediatric ND patients on exclusive enteral nutrition with a standard polymeric formula, no major complications occurred. Fourteen (37%) children had the gastrostomy tube changed to a skin-flush button device six months after the gastrostomy placement. In another retrospective study including 40 patients receiving a gastrostomy over 11 years [23], a total of 18 different complications were reported: 17 minor complications (peristomal site infection, mechanical complications, granulation tissue or scarring formation), and 1 major complication (gastrocolic fistula). Similarly, in the present study, only a few mild complications (i.e., peristomal site infection and granulation tissue) occurred. No major complications were reported. In a recent prospective, randomized, double-blind, placebo-controlled trial by Alessandri and colleagues [24], the administration of a single dose of intravenous co-amoxiclav showed a significant reduction in the rate of peristomal site infection from 21% in the placebo group to 5% in the treatment arm. Antibiotic prophylaxis to prevent peristomal site infection is currently recommended [25].

Physicians should emphasize the safety profile of gastrostomy placement while discussing indications and contraindications with families and caregivers in order to fight against barriers to gastrostomy placement (i.e., disinformation, psychological issues, parental anxiety, etc.). Notably, in our study, children started tube feeding only after an average of 2 years from the onset of the underlying disease.

One of the main findings of this study is that the normalization of the nutritional status (BMI Z-score > −2 SD) at 12 months was found to be negatively correlated with the disease duration before starting tube feeding. Patients in whom nutritional status did not normalize at 12 months had a significantly longer disease duration (before the start of tube feeding) than those in whom nutritional status normalized. Tube feeding is a life-saving treatment for pediatric patients with ND, but it is not yet widely used or is used late given the course of the underlying disease. The 2017 ESPGHAN guidelines recommend starting tube feeding early, even before the development of undernutrition [7]. Attainment of improved growth standards occurs more frequently in children treated early, i.e., before undernutrition becomes established [26].

We are aware of some limitations. First, this present study is an observational single-center, retrospective study, so recruited cases and clinical management are the confounding variables. Moreover, being a retrospective study, this may be confined only to our clinical findings and management. Consequently, our discussion is not a clinical guideline or consensus for the nutritional management of ND children. Second, some missing data are most likely to occur in this present study, and also, confounders might affect the differences between the factors. Third, regarding metabolic and genetic diseases, we did not differentiate between different conditions. The strength of this present study is that the indications of nutritional intervention, the timing and modality of enteral support, and the choice of formula are in conformity with current evidence-based guidelines and address a good-quality nutritional strategy for pediatric ND patients.

## 5. Conclusions

Tube feeding with commercially available enteral formulae is safe and effective in treating malnutrition in children with ND and should be started as early as possible for better outcomes. Future studies should include the option of utilizing the new food-based enteral formulae as ways to increase the indications and tolerance for enteral nutrition and decrease the risk of complications.

## Figures and Tables

**Table 1:** Baseline characteristics of enteral nutrition in the study population.

Variable	Total (*n* = 50)
Disease duration, years (mean ± SD) ^a^	2.6 ± 2.4
Enteral formula, *n* (%) ^b^	
Polymeric isocaloric	29 (58)
Semielemental isocaloric	17 (34)
Polymeric hypercaloric	3 (6)
Elemental	1 (2)
Homemade blended feed, *n* (%) *	
No	46 (92)
Yes ^c^	3 (6)
Enteral access, *n* (%)	
PEG	47 (94)
PEG-J	3 (6)
Regimen, *n* (%)	
Intermittent	38 (76)
Boluses	9 (18)
Continuous	3 (6)
Antireflux surgery, *n* (%)	
No	41 (82)
Yes	9 (18)

PEG, percutaneous endoscopic gastrostomy; PEG-J, PEG-jejunostomy; SD, standard deviation. ^a^ before starting tube feeding; ^b^ milk-based formulae; ^c^ in addition to the commercially available formula; * ≥1 missing data.

**Table 2:** Anthropometrics at baseline, 6, and 12 months (Wilcoxon test).

Variable, Median [IQR]	V0	V1	V2	*p* (V0 vs. V1)	*p* (V0 vs. V2)	*p* (V1 vs. V2)
Weight-for-age, Z-score	−3.5 [−5;−2]	−2.3 [−3.75;−1]	−2 [−3.33;−1]	<0.001 *	<0.001 *	0.073
BMI, Z-score	−1.35 [−3;−1]	−0.1 [−1.15;0.25]	1 [1.55;0.45]	0.004 *	0.064 *	0.042 *
MUAC, cm	14.75 [13;18]	14.8 [13;18]	16 [13.95;17.5]	0.285	0.285	0.109

BMI, body mass index; MUAC, mid-upper arm circumference; * statistically significant.

## Data Availability

The data that support the findings of this study are not openly available due to reasons of sensitivity and are available from the corresponding author upon reasonable request.
